# Development of Valid and Reliable Questionnaire to Evaluate Knowledge, Attitude, and Practices (KAP) of Lifestyle Medicine Domains

**DOI:** 10.3390/healthcare12161652

**Published:** 2024-08-20

**Authors:** Abeer Salman Alzaben, Mohammed Almansour, Hayat Saleh Alzahrani, Nouf Adnan Alrumaihi, Nesrain Mubarak Alhamedi, Nawaf Abdulaziz Albuhayjan, Sadeem Abdulaziz Aljammaz

**Affiliations:** 1 Department of Health Sciences, College of Health and Rehabilitation Sciences, Princess Nourah Bint Abdulrahman University, P.O. Box 84428, Riyadh 11671, Saudi Arabia; asalzaben@pnu.edu.sa; 2 Department of Medical Education, College of Medicine, King Saud University, P.O. Box 2925, Riyadh 11461, Saudi Arabia; 3 Family and Community Medicine Department, College of Medicine, Princess Nourah Bint Abdulrahman University, P.O. Box 84428, Riyadh 11671, Saudi Arabia; hsaalzahrani@pnu.edu.sa; 4 Academic Affairs, Saudi Commission for Health Specialty, Riyadh 11614, Saudi Arabia; n.alrumaihi@scfhs.org.sa; 5 Family Medicine Department, King Abdulaziz University Hospital, Jeddah 21589, Saudi Arabia; nalhamedi@kau.edu.sa; 6 College of Medicine, King Saud University, Riyadh 11461, Saudi Arabia; 438104166@student.ksu.edu.sa; 7 Community Health Sciences Department, College of Applied Medical Sciences, King Saud University, P.O. Box 4310, Riyadh 11491, Saudi Arabia; saljammaz@ksu.edu.sa

**Keywords:** lifestyle medicine, KAP questionnaire, validity, reliability, medical doctors

## Abstract

Lifestyle medicine (LM) should be incorporated as part of routine clinical work and medical education programs. Objective: To develop and test the validity and reliability of a questionnaire that measures the level of knowledge, attitude, and practice (KAP) of LM domains among medical trainees through practicing physicians. Methods: The KAP questionnaire sections covered the nine domains of LM. The validation process included face and content validity. A total of 151 individuals from the medical field residing in Saudi Arabia were recruited through a convenient sampling technique to participate in the study. Item response theory (IRT) was applied to validate the knowledge domain, while exploratory factor analysis (EFA) was used to assess attitude and practice. Cronbach’s alpha was performed to test the reliability of the three sections. Results: The questionnaire contained 37 items of knowledge, 45 attitudes, and 28 practice items. According to the IRT analysis, 27 items of knowledge were within the acceptable range of difficulty and discrimination. The EFA analysis resulted in 6 factors, including all the items in the attitude domain, and 4 factors, for a total of 27 items in the practice domain, with satisfactory factor loading (>0.4). The Cronbach’s alpha for the three domains was very high (≥0.88). Conclusions: The KAP questionnaire for LM is valid and reliable across a spectrum, from medical trainees to practicing physicians. This tool could serve as an instrument to evaluate and develop adequate educational programs for medical doctors.

## 1. Introduction

According to the World Health Organization (WHO), 41 million deaths each year are caused by noncommunicable diseases (NCDs), representing up to 74% of all deaths globally [1]. They are mostly resulting from unhealthy lifestyle behaviors, including tobacco use, stress exposure, fast food consumption, and alcohol use. All together, they promote the development of unhealthy changes at both metabolic and physiologic levels. These changes manifest as chronic diseases such as hypertension, obesity, diabetes, and dyslipidemia [2]. Based on the WHO’s recommendations, detection, screening, and treatment of NCDs, as well as palliative care, are key components of the response to NCDs [1]. In view of the vast amount of research analyzing investment in health, it is confirmed that prevention and control of NCDs are both essential to a cost-effective response strategy [3]. Thus, lifestyle medicine (LM) is an emerging field focusing on the evidence-based practice of assisting patients and their families to adopt and sustain behaviors that will improve their health and quality of life [4]. In fact, findings from research studies provide evidence supporting the clinical practice of using LM to treat and prevent NCDs, as stated by the American College of Lifestyle Medicine (2023) [5]. Even so, a study reported that most standard-of-care treatments and medical advice given to patients would not include a discussion on the root cause or prevention of NCDs [6]. Yet, seeking to reverse the incidence of these chronic diseases through combating the root causes could only be perceived as an important approach [5]. Medical professionals have recognized the effectiveness of lifestyle guidance in promoting patient health [7]. Within this context, a study confirmed that patients showed a tendency to consider with seriousness their doctor’s views since they perceive their opinion as reliable [8]. Moreover, previous studies reported that physicians have the capacity to influence patients’ lifestyle behaviors [4,9,10]. For example, Kreuter et al. (2000) revealed that the advice provided by the physician catalyzed the changes made by patients, such as smoking cessation, consuming less fat, or engaging in more exercise; such changes would happen before even receiving any intervention content on the same behavior [10]. Therefore, the American College of Lifestyle Medicine (2023) highlighted the importance of insisting on the new model in healthcare where physicians counsel patients on NCD prevention and treatment. Nevertheless, most physicians do not adopt routine advice on lifestyle behavior changes during medical clinic visits, which might have potential benefits for patients [11,12,13].

Health behaviors could greatly influence future health and well-being, especially among patients with chronic diseases [14]. To date, LM has been a relatively unknown approach for general physicians [6]. Taken together, it is evident that it should be a key component of interventional health promotion strategies. Consequently, this can be achieved by incorporating lifestyle medicine as part of routine clinical work and medical education programs established by primary care physicians, as they are at the forefront of health promotion and preventive medicine [15]. However, some barriers have been identified in the way of the widespread adoption of LM applicability. In their study, Huang et al. (2004) concluded that physicians have cited inadequate confidence and a lack of knowledge and skill as major barriers to counseling patients about lifestyle interventions [16]. Monti et al., 2023 recently revealed that despite an overall low level of awareness of LM, most physicians in their study expressed interest in a LM approach to health care [17].

Further research is needed to identify key interventions required to enhance the use of LM in routine physicians’ work. This could involve accurately evaluating the knowledge, attitudes, and practices (KAPs) of physicians regarding LM with its nine domains: nutrition, physical activity, health and wellness coaching, sleep health, emotional wellness, mindfulness, tobacco cessation, alcohol use, and weight management. A crucial component of appraising the level of KAP in the different domains of LM is the development of a valid and reliable questionnaire. Screening the literature, the only existing questionnaire evaluates certain domains of LM, as exemplified by the work of Chopra et al. [18]. These researchers developed a questionnaire that evaluated the impact of COVID-19 on lifestyle-related behaviors: eating habits, activity, and sleep behavior. Another questionnaire was validated by El Tantawi et al. [19]. Similarly, it was not complete and focused on the emotional wellness domain of LM only. The questionnaire assessed the impact of the COVID-19 pandemic on mental health and wellness. To the best of our knowledge, there is no comprehensive instrument designed to assess the degree of KAP pertaining to all of the domains of LM among medical students, residents, and physicians. The aim of this study was to develop and validate a questionnaire that measures the KAP of LM domains among both current medical doctors and medical students/trainee.

## 2. Materials and Methods

### 2.1. Study Participants

The study was conducted in Saudi Arabia. The inclusion criteria were medical practitioners such as medical students, residents, specialists, consultants, and faculty members, while excluding non-medical practitioners. Medical students were included as they are involved in operational medical teams. Additionally, building good KAP toward LM should start as early as possible to enhance the chances of the practice during their medical career.

A sample size of 151 participants was considered adequate to test the reliability of a KAP questionnaire based on the literature references (Tariq et al., 2022, Kennedy, 2022 and Mo et al., 2023), indicating that a sample size of at least 100 subjects was deemed sufficient to achieve reliable results for instrument reliability testing [20,21,22]. Hence, the authors increased the number by an additional 50% to ensure a more statistically robust evaluation of the KAP questionnaire’s reliability, as performed by Sosanya et al. (2023) [20,21,22,23]. Recruitment of participants was performed via a convenient sampling technique based on volunteer invitations. A link to the online questionnaire was distributed using a smart survey by WhatsApp, text message, or formal email sent by the Saudi Commission for Health Specialties. The sample was composed of 60% males and 40% females, with 33% being medical students and 66% being physicians (general physicians) and experts in LM living in Saudi Arabia. The data collected was anonymous and self-administered. Participants gave consent before responding to the questions by clicking on the “I agree” button. The time to complete the questionnaire ranged from 10 to 20 min. Ethical approval from the IRB was obtained from the Institutional Review Board at King Saud University (IRB number E-21-6373).

### 2.2. Development Process of the Questionnaire

The questionnaire was developed through the collaborative work of a group of experts who possess refined expertise and in-depth knowledge relevant to LM domains. The team included three family physicians, a specialist in LM, a specialist in medical education, and two registered nutritionists. Hence, these experts had overlapping expertise covering the nine LM domains. The seven experts were divided into three smaller teams to facilitate the development of questions with a focus on specific LM domains. The experts involved in developing the questionnaire were well-versed in both practical and theoretical aspects of lifestyle medicine. They carefully considered the depth of the questions and the interrelationships among the nine domains of lifestyle medicine in real-world contexts. This collaborative approach ensured consistency, coherence, and comprehensive coverage of all relevant aspects without redundancy. The developed questions covered nine domains of LM for each of the three sections of the KAP study. Once the questions were written, a peer-review process between the three teams was carried out to ensure complementarity, avoid redundancy, and enhance the overall quality and merit of the questionnaire. Developing the questionnaire followed the phases described below:

Literature search for any existing similar questionnaires, either comprehensive or contains some similar sections and/or domains in the main databases.

Agreement on the selected references for each section.

The selection of a pool of questions in each section of the KAP and each knowledge domain included more details.

Selecting the most appropriate 10–20 questions in each domain.

Cross-checking and peer-reviewing by other team members on the agreed questions to assure appropriateness.

Solving the disagreement on debatable questions was achieved by obtaining alternative wording, rephrasing, or paraphrasing the question.

Modifying the language or distractors length and clarity of questions as needed.

Final approval of the top five most appropriate questions in each domain in terms of the consensus between the 7 experts in addition to appropriateness, clarity, and testing. This was performed as per the existing literature and used references for each domain.

During the refinement of the questionnaire, one board member of American Lifestyle medicine was consulted for advice and recommendations because of his large and valuable expertise in the field as well as his interest in this research study.

Construct the questionnaire (Supplementary).

The generated questionnaire consisted of 47 questions distributed over three main sections: knowledge, attitude, and practice (see Supplementary). Each of the three main sections covered all nine domains of LM, which are: nutrition, physical activity, health and wellness coaching, sleep health, emotional wellness, mindfulness, tobacco cessation, alcohol use, and weight management (American College of Lifestyle Medicine, 2023) [5] (Table 1).

Section 1 Knowledge: this section consisted of 37 questions designed to assess the level of knowledge about LM domains. There were five questions for each of these six domains: nutrition, physical activity, sleep health, smoking cessation, alcohol control, and health and wellness coaching. Two questions were assigned for the weight management domain. The mindfulness and mental health well-being domains had overlapping questions, so they were combined to avoid redundancy and to reduce the length of the questionnaire. The references used to develop the questions in this section are Ifezulike et al., the UnAcademy website, and the American College of Lifestyle Medicine [24,25,26]. All the questions in the knowledge section were in a multiple-choice format with only one correct answer and three distractors. The correct answer had a score of 1 point, and each of the 3 incorrect answers had a score of 0. The minimum final score that could be obtained is 0 points, while the maximum is 37 points.

Section 2 Attitude: It contained five questions that were organized in such a manner that each question covered all nine domains, ensuring clarity, ease, coherence, and avoiding redundancy. Hence, each of the five questions had the format of a matrix of multiple choices with only one answer per row. The questions were developed and adopted from several references [27,28,29,30,31]. All the answers were designed on a 5-point Likert scale, as follows: 1 = Strongly disagree; 2 = Disagree; 3 = Neither agree nor disagree; 4 = Agree; 5-Strongly Agree. For the attitude section, there are a total of 45 questions (5 questions × 9 domains).

Section 3 Practice: In this section, four questions were included; they were designed based on several references [27,28,32,33,34,35,36]. Three of these questions were in a matrix of multiple-choice format with only one answer per row. These questions were designed to evaluate the extent of the implementation of LM domains in practice. The response choices were “always”, “frequently”, “sometimes”, “rarely”, and “never”. One multiple-choice question about the perceived barriers related to the practice of LM intervention. And the fourth question is about the level of confidence related to discussing LM interventions with patients. Possible answers were: 1 = No confidence, 2 = Low confidence, 3 = Neutral, 4 = Fair confidence, and 5 = High confidence. The total number of items in this section is 28.

### 2.3. Validity

After peer-reviewing and finalizing the questionnaire revision, it was evaluated by an external panel of seven members: six general physicians and one health educator. These experts were not involved in the process of questionnaire development. Each expert submitted an individual evaluation report containing scoring about the relevance of each questionnaire item in addition to comments or modification suggestions to improve the questionnaire. This step is key in the validation process, as proposed by Sousa [37]. The scoring system was based on a scale ranging from 1 to 5 on whether the questionnaire items were relevant or not. This scoring was performed for each of the 47 questionnaire items. If the average score obtained was <3, the questions were revised. The scoring obtained from the panel was also used to calculate the validity index of the questionnaire (face validity and content validity) [38].

### 2.4. Face Validity

To evaluate face validity, the researchers used the technique described by Ehrenbrusthoff et al. (2018) [39]. Thus, three main aspects were measured as follows:

1. Time to complete: this was calculated based on the score provided by the experts as per the appropriateness of time needed for filling out the questionnaire. The scoring was performed on a 0–10 scale, with 0 indicating “unacceptably long” and 10 “completely appropriate”.
2. Completeness of content: to assess this parameter, the experts responded by “Yes” or “No” whether they think that the questionnaire covered the most important aspects of KAP related to LM. The expert was asked to provide any complementary aspects to be added in case he/she answered by “No”.
3. Comprehensibility: regarding this parameter, the expert answered by “Yes” or “No” to whether the questions were sufficiently comprehensible. In case the chosen answer was no, the expert specified what item and why so that necessary modifications could be performed to improve the comprehensibility of the questionnaire.

These questions provided an in-depth understanding of the overall use of the questionnaire. As instructed by Janssens et al. (2017), the questionnaire underwent further revision as needed to ensure good quality in terms of content and linguistic structure of the items [40].

### 2.5. Content Validity

For each questionnaire item of the developed KAP questionnaire, each of the 7 panel expert members evaluated the relevance by giving a score of 1 (item not relevant) to 4 (item very relevant). Scores were categorized as follows: 3 and 4 were categorized = 1 (relevant), and scores of 1 and 2 = 0 (not relevant). Using the same scoring provided by the panel, calculations were performed to obtain the following: the Content validity Index for individual items (I-CVI), the Content Validity Index for the scale (S-CVI), the S-CVI/Average and the S-CVI/Universal agreement [41].

### 2.6. Item Response Theory

The unidimensionality of the knowledge domain with responses in dichotomous output as either the correct answer or the wrong answer requires two-parameter logistic item response theory (2-PL IRT) analysis to analyze the knowledge domain. An acceptable range of difficulty (−3 to +3) and discrimination (0.35 and 2.5) will serve as the cut-off values for the psychometric properties’ evaluation of the domain. The model fit of the knowledge scale was determined via the Cochran–Mantel–Haenszel test, commonly represented as M2. Item goodness-of-fit was assessed by standardized chi-square (S-X2) using a Monte Carlo simulation. Scale items with *p*-values greater than 0.05 reflected a good fit with the hypothetical model [42,43].

### 2.7. Exploratory Factor Analysis (EFA)

The construct validity of the attitude and practice domains of the questionnaire, due to their ordinal responses, was established by using exploratory factor analysis, the Kaiser–Meyer–Olkin (KMO) test, and Bartlett’s Test of Sphericity to check the sampling sufficiency for the factor analysis. EFA was used as the statistical method to identify the inter-relationships that exist among a large set of questionnaires. It is exploratory in nature to search for a possible underlying structure in the questionnaire and to extract items into constructed subscales called factors. The sample was considered adequate if the KMO value was more than 0.5 and Bartlett’s test was significant. The principal axis factoring method for the component extraction was used. Varimax rotation was applied to optimize the loading factor of each item on the extracted components. Components with Eigenvalues > 1 were retained as components using parallel analysis and scree plots. Items with a loading factor of more than plus or minus 0.3 were considered acceptable loading factors [23,44].

### 2.8. Reliability

The internal consistency of the questionnaire sub-scales derived from the construct validity was tested using Cronbach’s alpha coefficient. This coefficient ranges from 0 to 1. Large Cronbach’s alpha values indicate a high consistency of the questions [42]. A coefficient of >0.7 was used to consider good internal consistency.

Statistical analysis was performed using R software version 4.1.2 (R Foundation for Statistical Computing, Vienna, Austria). The level of significance was set at 0.05.

## 3. Results

### 3.1. Face Validity and Content Validity

The average scoring of the time needed to complete the questionnaire indicated that it was adequate, as reflected by the obtained value equal to 9.9 (±0.4) (based on a scale ranging from 0 to 10). For the completeness of the content and comprehensibility, there was full unanimity between the 7 experts (100% for both parameters). However, some minor comments were made, for example, avoiding the use of abbreviations like GP, which was replaced by General Practitioner, and REM, which was replaced by Rapid Eye Movement.

The values for the I-CVI were equal to 0.86 for 11 items and 1 for 36 items, while the CVI/Average was equal to 0.7. The S-CVI/Universal agreement value was 0.76, and the mean proportion of experts equaled 0.97. Feedback from the experts was mostly comments on the knowledge questions. Details are provided below:

### 3.2. Knowledge Section

Q9: Omar, a 45-year-old man, has been consistently playing football for the past four months. What best describes the stage of change he is at? And Q11 (knowledge): Which of the following statements most correctly describes the amount of nighttime sleep required by the age group?

Q22: Which of the following causes a rise in fat synthesis, blood vessel dilatation, stomach inflammation, and low blood sugar when consumed? Q27: Which of these statements most appropriately characterizes the impact of stress on work satisfaction, immune function, and surgical outcomes? And Q36: Which of the following statements is most accurate with regard to weight loss and exercise? As the stem contains several factors, the suggestion was to limit it to one factor per question.

### 3.3. Domain 5 on Alcohol in the Three Sections of the KAP

As per cultural and religious considerations in Saudi Arabia, this domain was commented on as not appropriate. However, this domain is considered one of 9 formally recognized domains in LM. Therefore, it was not removed. In addition, the questionnaire is foreseen to be used not only in Saudi Arabia but in other countries as well.

### 3.4. Attitude Section

There was one common comment for the 5 questions in this section, which was rephrasing the statement to make it more suitable from an “attitude” perspective. To this end, an introductory statement was added before the set of questions, directing the participant to respond as per his/her attitude regarding each statement.

### 3.5. Item Response Theory

Table 2 displays the analysis of the item response theory of the knowledge section. Due to high difficulty and discrimination, 10 items were removed from the initial 37 items on this scale. These 10 items are distributed as follows: 1 item in each of the domains Nutrition (K4), Sleep (K13), and Emotional wellness and wellbeing (K30), 3 in the domain of health and wellness coaching (K31–33), and 4 in the domain of Alcohol control (K21–25). No questions were removed from the domains of Physical activity, smoking cessation, and weight management. The retained questions in this section generally exhibited good psychometric characteristics and were approximate to, or within, the acceptable ranges of –3 to + 3 for difficulty and 0.35 to 2.5 for discrimination. Difficulty levels ranged from −2.89 for K7 to 1.72 for K3. The discrimination index for items ranged from 0.22 for K1 to 5.02 for K16. K2 had a lower discrimination level and K16 had a higher discrimination level, than the recommended cutoff but were retained because they met the criteria for other IRT parameters. All the items showed good fit (*p* > 0.05), except for K5, which had significant *p*-values. This question (K5) also retained good difficulty and discrimination values.

The results of the EFA for the attitude and practice domains are presented in Table 3 and Table 4, respectively. The KMO measure of sampling adequacy was 0.82 for the attitude domain and 0.87 for the practice domain, and the significance of Barlett’s test of sphericity was <0.001 for both domains. These findings indicated that EFA could be applied. For the attitude domain, a six-factor solution was obtained with a total of 45 items, and the total variance explained by the six factors was 76.3%. Only objects with factor loading ≥ 0.40 are shown. All attitude items were retained in the questionnaire (Table 3). For the practice domain, a four-factor solution was obtained with a total of 27 items, and the total variance explained by the four factors was 68.1%. Only items with a factor loading of ≥0.40 are shown. One item was removed (Table 4).

### 3.6. Reliability

The Cronbach’s alpha coefficients of knowledge, attitude, practice, and factor subscales are summarized in Table 5. They were all >0.70, indicating that the questionnaire had good internal consistency and reliability.

## 4. Discussion

To the best of the researchers’ knowledge, this study represents the first study describing the process of the conceptualization, design, and validation of a KAP questionnaire tailored for LM domains. Targeting physicians across various stages of their medical careers, this KAP instrument serves as a valuable assessment tool to obtain insights into and evaluate adherence to dispensing lifestyle change advice to patients. The integration of behavior modification guidance into physicians’ routine practice is increasingly recognized as a fundamental component of LM.

In this research study, diverse assessment methods were deployed to ensure the validity and reliability of the questionnaire, aiming to refine it to the most statistically sound version. The involvement of specialized raters at this critical stage served as a foundational step preceding the questionnaire’s testing by the target population [45]. Overall, the statistical analyses revealed that the questionnaire meets the requisite criteria of completeness, comprehensibility, and administration time suitability. Moreover, the mean expert CVI scale, calculated at 0.97, signifies an exceptional level of agreement among experts regarding the content’s relevance to the intended context of use and application [46].

Item Response Theory (IRT) is increasingly utilized in education and health research for modeling response data from assessments. Item difficulty concerns the relative ease or difficulty encountered by test takers when addressing specific items, as inferred from the proportion of accurate responses obtained. Conversely, item discrimination refers to an item’s ability to effectively differentiate between individuals with varying levels of achievement [47]. When applied to the knowledge section of the designed questionnaire, 27 out of 37 items exhibited acceptable and good psychometric properties based on discrimination and difficulty indices. The internal consistency of these retained 27 items, as measured by Cronbach’s alpha (α = 0.88), indicated satisfactory homogeneity within this section. However, discrepancies arose as ten items displayed values deviating from established cutoff points. One plausible explanation for these discrepancies lies in the unique cultural context of the study, particularly concerning alcohol control and health and wellness coaching domains. In Saudi Arabia, alcohol consumption is prohibited by religious norms and illegal by law [48], while health and wellness coaching remains a relatively novel concept not yet part of the healthcare routine [48]. Consequently, the applicability of IRT features may be compromised under these circumstances. Therefore, the researchers advocated for retaining these items in the final questionnaire version to maintain its comprehensive scope and original conception, as evidenced by previous studies retaining important items even if they did not fit with this proposed IRT model [42]. In the context of the present study, the content of the developed questionnaire is likely to be applied generally without concern about bias caused by different cultural or religious beliefs. Furthermore, the study’s findings may also be influenced by the small sample size (*n* = 151). Larger sample sizes are imperative for precise analysis and estimation of item properties, as well as for accurately assessing respondents’ abilities [49,50].

In the attitude section, exploratory factor analysis (EFA) revealed a two-factor structure as anticipated, explaining 76.3% of the total observed variance. All factor loadings surpassed 0.4, indicating strong associations between factors and questionnaire items [51]. Additionally, the reliability analysis of the attitude section showed a satisfactory Cronbach’s alpha value (α = 0.91), indicating internal consistency [52]. A similar approach was applied to the practice section, and the analysis yielded a well-fitting four-factor model (surpassing 0.4). Four items under the same question, related to the practice, were removed as their loading factor was <0.4. This led to strong reliability and internal consistency (Cronbach’s alpha value > 0.95). The EFA extracted four factors in this domain, explaining 68.1% of the total variance, surpassing the criterion of 50%.

In the attitude section, EFA revealed a six-factor structure as anticipated, explaining 76.3% of the total observed variance. The robust factor loadings, all surpassing 0.4, underscored the strong associations between factors and questionnaire items [51], validating the underlying constructs. Additionally, the reliability analysis demonstrated a high Cronbach’s alpha value (0.91), indicating excellent internal consistency within the attitude section [52]. Similarly, in the practice section, the analysis resulted in a well-fitting four-factor model, with all factor loadings exceeding 0.4. Notably, one item related to practice was removed due to its low loading factors (<0.4), ensuring the model’s integrity. This refinement contributed to the achievement of strong reliability and internal consistency (Cronbach’s alpha value = 0.95), enhancing the credibility of the measurement instrument [52]. The EFA extracted four factors in the practice domain, explaining a substantial 68.1% of the total variance, surpassing the commonly accepted criterion of 50%. This comprehensive explanatory power suggests that the identified factors effectively capture the multifaceted nature of practice behaviors in terms of LM. These findings not only validate the reliability and internal consistency of the research instrument but also underscore its utility in elucidating the complexities of practice-related constructs.

Regarding the removed item, “What is the level of practicing lifestyle medicine in your current practice”? The low loading observed in the EFA could indeed be attributed to sample heterogeneity. The diversity within the sample population may have introduced variability in responses, leading to weaker associations between certain items and the underlying factors [53]. In our study, this heterogeneity might stem from differences such as educational level, years of practice as a medical doctor, and frequency of practice delivering healthcare to patients, which can influence individuals’ perceptions and attitudes towards the surveyed practices.

One limitation of this study is the reliance on item response theory and exploratory factor analysis to assess the reliability and validity of the KAP questionnaire for LM practice. While these methods provide valuable insights into the underlying structure of the questionnaire and the relationships between variables, a confirmatory factor analysis (CFA) would offer further validation by testing the proposed factor structure against the data [54]. Therefore, it is recommended that future research include a CFA to confirm the factor structure identified in this study. This additional analysis would enhance the validity of the questionnaire and provide more robust evidence for its reliability and validity in assessing KAP related to LM among medical doctors. Another limitation is the small sample size, although it is considered sufficient, which could impact the generalizability of the results. In addition, the heterogeneity of a convenient sample, including medical students and practicing doctors, could introduce variability in responses, potentially affecting the reliability and validity of the KAP questionnaire on lifestyle medicine. This diversity may affect specific insights that could be more clearly delineated with level-specific questionnaires. However, by including a broad spectrum of medical counterparts, the study gains strength in assessing the robustness and generalizability of a comprehensive questionnaire across different stages of medical training and practice.

## 5. Conclusions

The study introduces a tailored questionnaire designed specifically to evaluate the KAP related to LM domains among medical doctors.

Utilizing this questionnaire offers the opportunity to gain insights into medical doctors’ KAP regarding lifestyle-related factors impacting health. Moreover, the questionnaire’s utility extends to the development of educational programs aimed at empowering healthcare professionals. Therefore, identifying areas of deficiency or misconception in any of the domains of LM and designing targeted educational interventions to bridge these gaps becomes feasible. This approach would foster continuous professional development and enhance the quality of care provided to the population, promoting adequate and effective behavior and lifestyle changes. Hence, it is highly recommended to reproduce further comprehensive studies in the field of KAP related to LM; this could provide deeper insights into how knowledge and attitudes influence healthcare practices regarding lifestyle interventions.

## Figures and Tables

**Table 1:** KAP questionnaire on LM domains.

Domain	No. of Items	Measurements	Response Options
General information		Socio-demographic, level of education, years of experience	Close-ended Multiple choice
Knowledge	37	Knowledge related to the 9 domains of lifestyle medicine	Yes/No 1 = correct answer 0 = Wrong answer
Attitude	45	Attitude towards including the 9 domains of lifestyle medicine	1 = strongly disagree, 2 = disagree, 3 = neutral, 4 = agree, 5 = strongly agree
Practice	28	Practices related to the 9 domains of lifestyle medicine	1 = never, 2 = rarely, 3 = sometimes, 4 = often, 5 = always Or 1 = No confidence, 2 = Low confidence, 3 = Neutral, 4 = Fair confidence and 5 = High confidence.

**Table 2:** Results of the IRT analysis in the knowledge section.

	a	b	χ2	df	*p* Value
K1	**0.22**	−1.85	4.816	10	0.903
K2	0.26	0.61	12.649	11	0.317
K3	1.03	1.72	8.717	5	0.121
K5	0.74	−2.08	13.539	6	0.035 *
K6	0.65	−1.14	8.282	10	0.601
K7	0.40	−2.89	5.317	7	0.621
K8	1.29	−1.93	0.734	3	0.865
K9	1.06	−0.12	9.921	10	0.447
K10	0.74	−0.05	10.577	10	0.391
K11	0.70	0.85	10.032	9	0.348
K12	0.96	−0.59	8.880	9	0.448
K14	0.35	−1.99	7.413	10	0.686
K15	1.82	0.55	6.245	6	0.396
K16	**5.02**	−0.98	0.059	1	0.808
K17	2.43	−0.62	1.067	5	0.957
K18	2.14	−1.00	5.376	4	0.251
K19	0.66	1.34	10.996	8	0.202
K20	1.29	0.34	5.305	7	0.623
K22	1.65	0.23	8.181	6	0.225
K26	4.02	−0.84	1.549	2	0.461
K27	0.47	−0.49	10.602	10	0.389
K28	1.66	0.12	8.511	6	0.203
K29	1.88	−0.04	5.468	6	0.485
K34	1.11	−0.25	7.900	9	0.544
K35	0.46	0.21	4.057	10	0.945
K36	0.53	−0.31	7.607	10	0.667
K37	1.37	−1.43	3.662	5	0.599

* Significant at *p* < 0·05, M2 = 362.244, *p* = 0.070, Comparative Fit Index = 0.927, Tucker–Lewis Index = 0.932, Root Mean Square Error of approximation = 0.043. a discrimination, b difficulty, IRT item response theory, χ2 chi-square; (0.22) lowest discrimination level, (5.02) highest discrimination level and (0.035) significant *p*-value.

**Table 3:** Exploratory factor analysis of items in attitude using principal axis extraction with varimax rotation.

Items	Loadings					
	Factor1	Factor2	Factor3	Factor4	Factor5	Factor6
A1.1. Weight management						0.838
A1.2. Nutrition						0.833
A1.3. Physical activity						0.880
A1.4. Smoking cessation						0.860
A1.5. Alcohol control						0.823
A1.6. Sleep Health						0.851
A1.7. Emotional Wellness						0.824
A1.8. Mindfulness						0.791
A1.9. Health and Wellness Coaching						0.775
A2.1. Weight management				0.883		
A2.2. Nutrition				0.827		
A2.3. Physical activity				0.869		
A2.4. Smoking cessation				0.845		
A2.5. Alcohol control				0.777		
A2.6. Sleep Health				0.728		
A2.7. Emotional Wellness					0.610	
A2.8. Mindfulness					0.580	
A2.9. Health and Wellness Coaching					0.576	
A3.1. Weight management		0.824				
A3.2. Nutrition		0.848				
A3.3. Physical activity		0.875				
A3.4. Smoking cessation		0.792				
A3.5. Alcohol control		0.812				
A3.6. Sleep Health		0.886				
A3.7. Emotional Wellness		0.819				
A3.8. Mindfulness		0.855				
A3.9. Health and Wellness Coaching		0.882				
A4.1. Weight management			0.799			
A4.2. Nutrition			0.840			
A4.3. Physical activity			0.878			
A4.4. Smoking cessation			0.878			
A4.5. Alcohol control			0.884			
A4.6. Sleep Health			0.784			
A4.7. Emotional Wellness			0.632			
A4.8. Mindfulness					0.648	
A4.9. Health and Wellness Coaching					0.643	
A5.1. Weight management	0.785					
A5.2. Nutrition	0.798					
A5.3. Physical activity	0.868					
A5.4. Smoking cessation	0.876					
A5.5. Alcohol control	0.870					
A5.6. Sleep Health	0.836					
A5.7. Emotional Wellness	0.764					
A5.8. Mindfulness	0.558					
A5.9. Health and Wellness Coaching	0.597					

KMO measure of sampling adequacy = 0.82, Bartlett’s sphericity test: Chi-squared = 7266.753, degree of freedom: 990, significance > 0.001.

**Table 4:** EFA of items in practice and analysis of reliability.

Items	Loadings			
	Factor1	Factor2	Factor3	Factor4
P3.1. General lifestyle	0.716			
P3.2. Weight management	0.844			
P3.3. Healthy food	0.779			
P3.4. Physical activity	0.825			
P3.5. Smoking cessation				0.625
P3.6. Alcohol control				0.722
P3.7. Sleep Health		0.558		
P3.8. Emotional Wellness		0.645		
P3.9. Mindfulness		0.848		
P3.10. Health and Wellness Coaching		0.768		
P4.1. Weight management			0.743	
P4.2. Healthy food			0.845	
P4.3. Physical activity			0.790	
P4.4. Smoking cessation				0.545
P4.5. Alcohol control				0.721
P4.6. Sleep Health			0.727	
P4.7. Emotional Wellness			0.627	
P4.8. Mindfulness		0.734		
P4.9. Health and Wellness Coaching		0.749		
P5.1. Coach/discuss how to change lifestyle	0.689			
P5.2. Prescribe exercise	0.661			
P5.3. Advise patients to “manage their weight”	0.768			
P5.4. Advise appropriate diet	0.726			
P5.5. Discuss stress management	0.615			
P5.6. Provide lifestyle educational materials		0.535		
P5.7. Refer to specialist for further lifestyle interventions		0.410		
P5.8. Refer to support groups		0.649		

KMO measure of sampling adequacy = 0.87, Bartlett’s sphericity test: Chi-squared = 2986.624, degree of freedom: 378, significance < 0.001.

**Table 5:** Analysis of reliability (internal consistency–Cronbach’s alpha) of KAP domains.

		Cronbach’s Alpha	No. of Items
Knowledge		0.88	27
Attitude	Overall attitude	0.91	45
	Factor1	0.95	9
	Factor2	0.96	9
	Factor3	0.95	7
	Factor4	0.94	6
	Factor5	0.88	5
	Factor6	0.96	9
Practice	Overall practice	0.95	27
	Factor1	0.94	9
	Factor2	0.92	9
	Factor3	0.90	5
	Factor4	0.82	4

## Data Availability

Available upon request.

## Supplementary Materials

The following supporting information can be downloaded at: https://www.mdpi.com/article/10.3390/healthcare12161652/s1, Questionnaire to evaluate knowledge, attitude and practices of lifestyle medicine domains.
