# Supplementary material for: Development of Valid and Reliable Questionnaire to Evaluate Knowledge, Attitude, and Practices (KAP) of Lifestyle Medicine Domains
Source: Healthcare (Basel). 2024 Aug 20;12(16):1652. doi: 10.3390/healthcare12161652 (PMC11353863; doi:10.3390/healthcare12161652)
Supplement: Supplementary file 1 [file healthcare-12-01652-s001.zip › healthcare-3063095-supplementary.pdf]

Questionnaire to evaluate knowledge, attitude and practices of lifestyle medicine domains

I. **Knowledge of lifestyle medicine domains** (*Correct answer (highlighted in yellow) = 1 score, Incorrect answer = 0 score*) ***(In red, the questions suggested to be removed based on the IRT analysis, kindly refer to the manuscript results and discussion sections)***

**Domain 2: Nutrition**	
**Q1: Which of the following statements is the most accurate when considering a plant-based diet?**	
It typically lowers HDL cholesterol	0
It typically lowers LDL cholesterol	1
It typically lowers total cholesterol	0
No difference in cholesterol compared to other diets	0
**Q2: When considering fats, which of the following statements is the most accurate?**	
Harmful saturated fats should be eaten in moderate amounts	0
Trans fats should be completely eliminated	1
Trans fat foods should be minimized	0
Trans fats should be generously prescribed	0
**Q3: Ali, a 46-year-old man, has been advised to increase his fiber intake by his general practitioner to help with bowel function. Which of the following best describes the amount of fiber per day he should ideally aim for?**	
15 g	0
25 g	0
20 g	0
35 g	1
**Q4: Ahmed is a 50 years old man and has elevated total cholesterol. He wishes to reduce the fat content of his diet. Which of the following is the best approximation of the percentage of total energy requirements from fat?**	
5–10%	0
10–15%	0
15–20%	0
25–30%	1
**Q5: A patient asked why dark chocolate is often described as more beneficial than milk chocolate. Which of the following is the most accurate explanation?**	

It contains saturated fatty acid that is more likely to convert to cholesterol esters	0
It contains more sugar	0
It is associated with a lower HDL level	0
It is associated with a lower LDL cholesterol compared to other saturated fats	1
**Domain 3: Physical activity**	
**Q6: Which of the following best describes the effects of warming up prior to physical activity (PA)?**	
Decrease range of motion at a joint, increase physical work capacity, increase the rate of energy release from the body	0
Increase range of motion at a joint, decrease physical work capacity, increase the rate of energy release from the body	0
Increase range of motion at a joint, increase physical work capacity, decrease the rate of energy release from the body	0
Increase range of motion at a joint, increase physical work capacity, increase the rate of energy release from the body	1
**Q7: Which of the following best describes examples of aerobic exercises?**	
Bicycling, stretching, swimming	0
Bicycling, running, swimming	1
Bicycling, running, stretching	0
Running, stretching, swimming	0
**Q8: Which of the following best describes the benefits of strength training?**	
Improves heart muscle functioning, increases fatigue, increases muscle mass	0
Improves heart muscle functioning, reduces fatigue, increase muscle mass	1
Improves heart muscle functioning, reduces fatigue, reduces muscle mass	0
Reduces heart muscle functioning, reduces fatigue, increase muscle mass	0
**Q9: Omar, a 45-year-old man, has been consistently playing football for the past four months. What best describes the stage of change he is at?**	
Maintenance stage	1
Action stage	0
Preparation stage	0
Contemplation stage	0
**Q10: Which of these most accurately describes the recommendations regarding physical activity (PA) in pregnancy?**	

In normal healthy women, the recommendation is at least 75 minutes of moderate-intensity aerobic activity per week	0
Guidelines generally rule out sports with risks of falls, trauma, or collisions	1
All pregnant women once confirmed pregnant should avoid PA that involves lying on their back	0
Scuba diving is usually recommended	0
**Domain 6: Sleep Health**	
**Q11: Which of the following statements most correctly describes the amount of nighttime sleep required by the age group?**	
Infants require 8 - 10 hours of sleep	0
Newborns require 10 - 12 hours of sleep	0
School-age children require 7 - 8 hours of sleep	0
Toddlers require 11 - 14 hours of sleep	1
**Q12: Which of the following best describes an action in sleep hygiene for sleep improvement?**	
Decrease bedtime peripheral cutaneous vasodilation by wearing cooling socks	0
Establish sleep time but wake time can differ depending on your plan for the day	0
Have power naps of no more than 30 minutes	1
Use the alarm clock as the room light	0
**Q13: Concerning the activities of the brain during sleep, which of the following best represents the stages of sleep?**	
Stage 5: Rapid Eye Movement (REM) sleep	1
Stage 4: Deep sleep with a combination of theta and delta waves	0
Stage 3: Deepest sleep, theta waves disappear, and there are only delta waves	0
Stage 2: Light sleep characterized by delta waves only	0
**Q14: Which of the following conditions is the best example of a lifestyle- related sleep disorder?**	
Sleepwalking	0
Sleep talking	0
Obstructive sleep apnoea	1
Nightmares	0
**Q15: In advising a new patient seen about sleep hygiene, which of the following will you recommend that they best avoid?**	

Inconsistent sleep- wake pattern	1
Increase moderate physical activity to about five times a week	0
Removal of television from the bedroom	0
Use of meditation every night to aid sleep	0
**Domain 4: Smoking cessation**	
**Q16: Which of these are true for 'second- hand smoking'?**	
It is the smoke that fills restaurants, offices, or other enclosed spaces when people burn tobacco products	1
Second- hand smoke does not affect children	0
Second- hand smoke does not cause serious cardiovascular and respiratory diseases	0
There is a safe level of exposure to second- hand tobacco smoke	0
**Q17: Which of the following risks associated with smoking tobacco is most accurate?**	
Smoking causes about 50 % of lung cancers	0
Smoking does not cause cancer of the mouth	0
Smoking increases risk of developing more than 50 serious health conditions	1
Smoking- related illnesses do not cause irreversible long- term damage to health	0
**Q18: Which of the following has been shown to be the most effective for tobacco cessation?**	
Acupuncture	0
Counselling	0
Counselling and Medication together	1
Medication	0
**Q19: Which of the following is unlikely to be a step in the counseling for tobacco cessation?**	
Advice to quit	0
Agree quit goals	1
Ask about tobacco use	0
Assess readiness to make a quit attempt	0
**Q20: Sami, a 43- year- old man, whose wife recently gave birth, is keen to stop smoking because of his baby son. He currently smokes 20 cigarettes a day. Which of the following would be the least appropriate treatment to initiate?**	
Clonidine	1
Nicotine replacement patch	0

Varenicline	0
Nicotine replacement Lozenges	0
**Domain 5: Alcohol control**	
**Q21: Regarding the Alcohol Use Disorders Identification Test (AUDIT), which of the following is the most accurate?**	
AUDIT has lower sensitivity and specificity than other diagnostic screening instruments such as the CAGE and MAST	0
It was developed by the World Health Organization for use in clinical settings to screen for people at risk of developing alcohol problems	1
The instrument consists of 9 items intended to cover 3 domains of hazardous drinking (quantity and frequency of use, dependence symptoms and other problems from alcohol use)	0
Nicotine replacement Lozenges	0
**Q22: which of the following causes a rise in fat synthesis, blood vessel dilatation, stomach inflammation and low blood sugar when consumed?**	
Drug addiction & tobacco	0
Drug addiction	0
Tobacco	0
Alcohol	1
**Q23: Which of the following is the result of the appearance of fatty liver syndrome?**	
Excess synthesis of the fat from fatty acid.	0
Synthesis of fat, fatty acids, & glycerol.	0
Synthesis of the fat from amino acid.	0
Synthesis of the fat from alcohol.	1
**Q24: Which of the following is the withdrawal symptom of the alcohol consumption?**	
Delirium	0
Vomiting & nausea	1
Patchy & swollen face	0
None of these	0
**Q25: Which of the following is the consequence of addiction to alcohol?**	
Hypertension, psychosis and fatty liver syndrome.	0
Vitamin deficiency, ulcers, cardiovascular diseases and all types of mental illness.	0
Hypertension, fatty liver syndrome and cardiovascular diseases.	1
All of the above	0

Domain 7/8: Emotional Wellness and Mindfulness	
**Q26: Which of these statements best illustrates the relationship between stress and the drive to eat?**	
Stress has no impact on eating habits or choice of food at all	0
Stress enhances healthy meal choices due to release of adrenaline	0
Stressed people more frequently eat nutritious food such as fruits and vegetables	0
Stressed people more frequently eat palatable non-nutritious food such as fried chips and soda	1
**Q27: Which of these statements most appropriately characterized the impact of stress on work satisfaction, immune function, and surgical outcomes?**	
Perceived patient complexity has been linked to increasing primary care physicians work satisfaction	0
Physicians feel well-equipped to provide the socio-economic interventions patients truly need, contributing to increased stress	0
Stress reduction techniques in elderly improve immune function with less tendency for catching flu	1
Surgical patients with higher stress levels and poor coping skills have better outcomes	0
**Q28: Which of these statements defines mindfulness?**	
A space of awareness in which one can witness and investigate the activities of their neighbor's mind and body	0
Being judgmental on purpose and striving with one's spouse	0
Deep in our thoughts and unable to notice how those thoughts are driving our emotions and behavior	0
Developing the potential to experience each moment with serenity and clarity	1
**Q29: Which of these bests characterized some of the benefits of mindfulness-based stress reduction (MBSR)?**	
Erodes psychological hardiness	0
Greater energy and enthusiasm for life	1
Increased ability to argue	0
Loneliness among the elderly	0
**Q30: Which of these options best characterizes personal therapeutic lifestyle change intervention?**	
Counselling session with behavior therapist	0

Pharmacotherapy with antidepressants	0
Recreation and relaxation with family	1
Starvation and time in nature	0
**Domain 9: Health and Wellness Coaching**	
**Q31: You are working with patient on a goal and patient's confidence level in succeeding is 8/10, What should be your next steps with this patient?**	
This confidence level is associated with success, so no adjustment of the goal is needed	1
This confidence level is unacceptable, and the goal should be avoided	0
This confidence level is close to an ideal confidence level for success and may require some adjustment of the goal	0
This confidence level is not an important predictor of behavior change	0
**Q32: You are coaching a patient for health behavior change to help build motivation. Which of the following you should do?**	
Provide detailed information about each stage of change that the patient will go through	0
Assume what the patient already knows, and give them only supplemental information Avoid all or nothing thinking	0
Start with the area in which the patient has the least confidence	1
All of the above	0
**Q33: You are using reflection in your patient interaction and say to your patient, " I hear you saying that you need to exercise but that you have little to no time." This is an example of what type of reflection?**	
Simple	0
Double-sided	1
Summarizing	0
Empathy	0
**Q34: The 5 transtheoretical stages of change in order are:**	
Precontemplation, contemplation, progress, action, maintenance	0
Precontemplation, contemplation, action, maintenance, progress	0
Contemplation, action, maintenance, progress, redo	0
Precontemplation, contemplation, preparation, action, maintenance	1
**Q35: An action plan must include all of the following Except:**	
It must be evidence-based, achievable and specific to the patient	0
It must incorporate only the patient rather than the patient and his/her support system (i.e., family and friends)	1

It must be written down, and a copy must be provided to the patient as well as placed in the chart	0
It will have more success if it's written down as a prescription and given to the patient followed by an agreement-binding handshake	0
**Domain 2: Weight management**	
**Q36: Which of the following statements is most accurate with regard to weight loss and exercise?**	
People always need more than the recommended 150 minutes physical activity per week to lose weight	0
People vary in the amount of physical activity they need to achieve and maintain a healthy weight	1
High levels of physical activity alone are required to lose weight	0
More than 300 minutes per week of physical activity are the main strategy to lose body weight	0
**Q37: The body mass index (BMI) is a useful clinical tool for diagnosing obesity. At what BMI level can the diagnosis of obesity be made?**	
BMI>= 18	0
BMI>= 23	0
BMI>= 30	1
None of the above	0

II. Attitude towards lifestyle medicine domains (*Scoring: Negative statement (Q1 and Q3): Strongly Disagree = 5 – Strongly Agree 1, Positive statement Strongly Disagree = 1 – Strongly Agree 5*)

	**Strongly Disagree**	**Disagree**	**Neither Agree nor Disagree**	**Agree**	**Strongly Agree**
**Q1: It is Not an efficient use of my time to counsel patients on:**					
Domain 1: Weight management					
Domain 2: Nutrition					
Domain 3: Physical activity					
Domain 4: Smoking cessation					
Domain 5: Alcohol control					
Domain 6: Sleep Health					
Domain 7: Emotional Wellness					

Domain 8: Mindfulness					
Domain 9: Health and Wellness Coaching					
**Q2: Advice from a physician is one of the best ways to help people start to change in:**					
Domain 1: Weight management					
Domain 2: Nutrition					
Domain 3: Physical activity					
Domain 4: Smoking cessation					
Domain 5: Alcohol control					
Domain 6: Sleep Health					
Domain 7: Emotional Wellness					
Domain 8: Mindfulness					
Domain 9: Health and Wellness Coaching					
**Q3: Counseling patients about the following is very frustrating:**					
Domain 1: Weight management					
Domain 2: Nutrition					
Domain 3: Physical activity					
Domain 4: Smoking cessation					
Domain 5: Alcohol control					
Domain 6: Sleep Health					
Domain 7: Emotional Wellness					
Domain 8: Mindfulness					
Domain 9: Health and Wellness Coaching					
**Q4: It is my responsibility as a physician to counsel my patients on:**					
Domain 1: Weight management					
Domain 2: Nutrition					
Domain 3: Physical activity					
Domain 4: Smoking cessation					
Domain 5: Alcohol control					
Domain 6: Sleep Health					
Domain 7: Emotional Wellness					
Domain 8: Mindfulness					

Domain 9: Health and Wellness Coaching					
**Q5: Physicians should get specific training on techniques to help patients with:**					
Domain 1: Weight management					
Domain 2: Nutrition					
Domain 3: Physical activity					
Domain 4: Smoking cessation					
Domain 5: Alcohol control					
Domain 6: Sleep Health					
Domain 7: Emotional Wellness					
Domain 8: Mindfulness					
Domain 9: Health and Wellness Coaching					

III. Practice related to lifestyle medicine domains (*Scoring: 1 to 4 based on the practice for Q1, and 1-5 based on frequency for Q3 and Q5, and level of confidence for Q4. Q2 should not be included in the reliability test)*

**Q1: What is the level of practicing lifestyle medicine in your current practice? (TO BE DELETED???)**	
I have neither recommended nor practiced it	1
I have recommended it	2
I have referred to it	3
I have practiced it	4
**Q2: From your own experience, what are the barriers regarding practicing lifestyle medicine?(multiple choices allowed) (TO BE DELETED???)**	
Limited time	To **NOT** to be I included in the reliability analysis (it will be a treated as a separate question as it deals with BARRIERS
Lack of incentives	
Lack of knowledge about the field	
Lack of treatment skills	
Lack of materials	
Lack of patient motivation	
Perceived patients' poor compliance	

Lack of support services					
Other (please specify):					
**Q3: How frequent do you talk to your patients regarding the following lifestyle medicine topics?**	**Never**	**Rarely**	**Sometimes**	**Usually**	**Always**
General lifestyle					
Domain 1: Weight management	1	2	3	4	5
Domain 2: Nutrition	1	2	3	4	5
Domain 3: Physical activity	1	2	3	4	5
Domain 4: Smoking cessation	1	2	3	4	5
Domain 5: Alcohol control	1	2	3	4	5
Domain 6: Sleep Health	1	2	3	4	5
Domain 7: Emotional Wellness	1	2	3	4	5
Domain 8: Mindfulness	1	2	3	4	5
Domain 9: Health and Wellness Coaching	1	2	3	4	5
**Q4: How confident are you in discussing the following lifestyle interventions with your patients?**	**No confidence**	**Low confidence**	**Neutral**	**Fair confidence**	**High confidence**
Domain 1: Weight management	1	2	3	4	5
Domain 2: Nutrition	1	2	3	4	5
Domain 3: Physical activity	1	2	3	4	5
Domain 4: Smoking cessation	1	2	3	4	5
Domain 5: Alcohol control	1	2	3	4	5
Domain 6: Sleep Health	1	2	3	4	5
Domain 7: Emotional Wellness	1	2	3	4	5
Domain 8: Mindfulness	1	2	3	4	5
Domain 9: Health and Wellness Coaching	1	2	3	4	5

Q5: How often do you practice the following lifestyle interventions on your patients?	Never	Rarely	Sometimes	Usually	Always
Coach/discuss how to change lifestyle	1	2	3	4	5
Prescribe exercise	1	2	3	4	5
Advise patients to "manage their weight"	1	2	3	4	5
Advise appropriate diet	1	2	3	4	5
Discuss stress management	1	2	3	4	5
Provide lifestyle educational materials	1	2	3	4	5
Refer to specialist for further lifestyle interventions (dietitian, physical coach, sleep specialist, psychologist, health coach..etc)	1	2	3	4	5
Refer to support groups (e.g. Wl Watchers, etc..)					
